# Clinical Pharmacology in Old Persons

**DOI:** 10.6064/2012/723678

**Published:** 2012-07-28

**Authors:** Paul A. F. Jansen, Jacobus R. B. J. Brouwers

**Affiliations:** Expertise Centre Pharmacotherapy in Old Persons, University Medical Centre Utrecht, B05.256, P.O. Box 85500, 3508 GA Utrecht, The Netherlands

## Abstract

The epidemiological transition, with a rapid increase in the proportion in the global population aged over 65 years from 11% in 2010 to 22% in 2050 and 32% in 2100, represents a challenge for public health. More and more old persons have multimorbidities and are treated with a large number of medicines. In advanced age, the pharmacokinetics and pharmacodynamics of many drugs are altered. In addition, pharmacotherapy may be complicated by difficulties with obtaining drugs or adherence and persistence with drug regimens. Safe and effective pharmacotherapy remains one of the greatest challenges in geriatric medicine. In this paper, the main principles of geriatric pharmacology are presented.

## 1. Introduction

**Figure 1:**
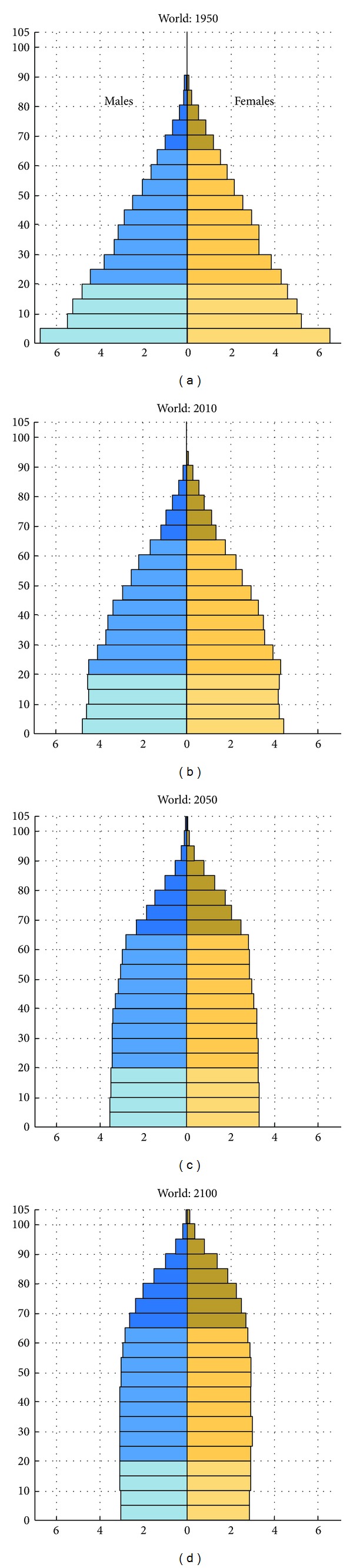
Increase in life expectancy from 1950 until 2100. Population by age groups and sex expressed as percentage of total population [2].

The worldwide population, within the age group 65 years and older has increased rapidly in the last century and a further increase is expected (Figure 1). The proportion of the global population over 65 years old increases from 11% in 2010 to 22% in 2050 and 32% in 2100 [1, 2]. The proportion aged above 80 years in western Europe will increase from 4% in 2010 up to 10% in 2050.

The ageing of the world's population is the result of several factors: installation of sewers and improvement of potable water, improvement of quality of food and preservation of food, better housing, education, more attention for physical condition, and developments in medical sciences [3]. Prevention and treatment of infectious and cardiovascular diseases and development of anaesthesiology medicines and technics have, amongst others, contributed considerably to the increase in life expectancy. An epidemiological transition in the leading causes of death, from infectious disease and acute illness to noncommunicable chronic diseases and degenerative illnesses is happening. Developed countries in North America, Europe, and the Western Pacific already underwent this transition, and other countries are at different stages of progression. The epidemiological transition, combined with the increasing number of older people, represents a challenge for public health. More and more old persons have multimorbidities and are treated with five medicines or more. In advanced age, the pharmacokinetics and pharmacodynamics of many drugs are altered. In addition, pharmacotherapy may be complicated by difficulties with obtaining drugs or complying with drug regimens. Safe and effective pharmacotherapy remains one of the greatest challenges in geriatric medicine. In this paper, the principles of geriatric pharmacology are presented.

## 2. Age-Related Changes in Pharmacokinetics

With increasing age and because of change in body weight, several changes in pharmacokinetics are present in many elderly people. Especially changes in volume of distribution and renal clearance are of clinical importance [4].

### 2.1. Drug Absorption

Pharmacokinetic studies on the effect of ageing on drug absorption have provided conflicting results. Several studies have not shown age-related differences in absorption rates for different drugs [5]. However, other studies have shown an increased absorption of, for example, levodopa. For drugs absorbed by passive diffusion there is low grade evidence for age-related changes. In general no adaptation of the dose is needed because of the ageing process.

### 2.2. First-Pass Metabolism and Bioavailability

There is a reduction in first-pass metabolism with advancing age. This is probably due to a reduction in liver mass and, for high clearance drugs, the consequential reduction in blood flow. The bioavailability of drugs which undergo extensive first-pass metabolism such as opioids and metoclopramide, can be significantly increased. For these drugs a low start dose is advised. By contrast, the first-pass activation of several prodrugs, such as the angiotensin-converting-enzyme-(ACE-) inhibitors enalapril and perindopril, might be slower or reduced [6]. However, this is not clinically relevant due to the chronic usage.

### 2.3. Drug Distribution in the Body

Significant changes in body composition occur with advancing age, such as a progressive reduction in the proportion of total body water and lean body mass. This results in a relative increase in body fat. Hydrophilic drugs tend to have smaller volume of distribution (V) resulting in higher serum levels in older people (e.g., gentamicin, digoxin, lithium, and theophylline). The consequence may be that the loading dose should be lower than in young adults. The reduction in v for water-soluble drugs tends to be balanced by a larger reduction in renal clearance (CL), with a smaller effect on elimination half life (*t* ^1^/_2_el__). By contrast, lipophilic drugs (e.g., benzodiazepines, morphine, and amiodarone) have a lower water solubility so their V increases with age. The main effect of the increased V is a prolongation of half-life. Increased V and   *t* ^1^/_2_el__ have been observed for drugs such as diazepam, thiopental and lidocaine. The consequence is that old patients may have long-lasting effects and adverse effects after cessation of the therapy [4].

### 2.4. Protein Binding

Acidic compounds (e.g., diazepam, phenytoin, warfarin, acetylsalicylic acid) bind mainly to albumin whereas basic drugs (e.g., lidocaine, propranolol) bind to alpha-1 acid glycoprotein. Although no substantial age-related changes in the concentrations of both these proteins have been observed, albumin is commonly reduced in persons with malnutrition, cachexia, or acute illness whereas alpha-1 acid glycoprotein is increased during acute illness. The main factor which determines the drug effect is the free (unbound) concentration of the drug. Although plasma protein binding changes might theoretically contribute to drug interactions or physiological effects for drugs that are highly protein-bound, its clinical relevance is limited for most of the drugs [13]. However, for some medicines, for example, phenytoin, drug effects may be enhanced and more ADR could be seen with low albumin concentrations [14].

### 2.5. Drug Clearance

#### 2.5.1. Liver

Drug clearance by the liver depends on the capacity of the liver to metabolize the drug from the blood passing through the organ (hepatic extraction ratio) and hepatic blood flow. Drugs can be classified into three groups according to their extraction ratio (*E*): high (*E* > 0.7, such as dextropropoxyphene, lidocaine, pethidine, and propranolol), intermediate (*E* 0.3–0.7, such as acetylsalicylic acid, codeine, morphine, and triazolam), and low extraction ratio (*E* < 0.3, such as carbamazepine, diazepam, phenytoin, theophylline, and warfarin). When *E* is high, the clearance is rate-limited by blood flow. When *E* is low, changes in blood flow produce little changes in clearance. Therefore, the reduction in liver blood flow with ageing mainly affects the clearance of drugs with a high extraction ratio. Of much greater importance is the reduction in liver volume up to as much as 30% across the adult age range.This results in a reduction in clearance of a similar magnitude [15]. Several studies have shown significant age-related reductions in the clearance of many drugs metabolised by phase-1 pathways in the liver. These involve reactions such as oxidation and reduction. The amount of total Cytochrome P 450-metabolizing enzymes (CYP) is decreased in patients over 70 years of age with about 30% [16]. By contrast, phase-2 pathways (e.g., glucuronidation) do not seem to be significantly affected [15]. However, in general the reduction in hepatic clearance is not of clinical relevance and dose reduction is not needed.

#### 2.5.2. Kidney

The age-related reduction in glomerular filtration rate affects the clearance of many drugs such as water-soluble antibiotics, diuretics, digoxin, water-soluble beta-blockers, lithium, nonsteroidal anti-inflammatory drugs, and newer anticoagulant drugs like dabigatran and rivaroxaban. The clinical importance of such reductions of renal excretion is dependent on the likely toxicity of the drug. Drugs with a narrow therapeutic index like aminoglycoside antibiotics, digoxin, and lithium are likely to have serious adverse effects if they accumulate only marginally more than intended. In elderly patients the serum creatinine may be within the reference limits, while renal function is markedly diminished. Estimation of the creatinine clearance or glomerular filtration rate with the Cockcroft and Gault or the Modification of Diet in Renal Disease (MDRD) equations may be helpful. However these methods are not yet validated in frail elderly patients, therefore one should be careful when using these equations [17–19].

## 3. Age-Related Changes in Pharmacodynamics

Studies of drug sensitivity require measurement of concentrations of drug in plasma, as well as measurement of drug effects. Pharmacodynamics are determined by concentrations of the drug at the receptor, drug receptor interactions (variations in receptor number, receptor affinity, second messenger response, and cellular response), and homeostatic regulation. Few data are available on pharmacodynamic differences in very old persons [20]. Some important pharmacodynamic age-related changes are illustrated in Table 1.

### 3.1. Anticoagulants

A number of studies have shown that the frequency of bleeding events associated with anticoagulants therapy and response to warfarin increase with age [20, 21]. There is evidence of a greater inhibition of synthesis of activated vitamin K-dependent clotting factors at similar plasma concentrations of warfarin in elderly compared to young patients. If vitamin K-antagonists (VKAs) are monitored carefully, age in itself is not a contraindication for treatment and as presented in an Italian study in the very old, the VKA's have acceptable low rates of bleeding incidents [22]. Concerning the new anticoagulants, dabigatran, rivaroxaban and apixaban, prescribers should be aware of the differences between well-controlled trials and daily practice, especially concerning adverse drug events (ADEs). If prescribed to the elderly, appropriate doses should be used [23].

### 3.2. Cardiovascular Drugs

#### 3.2.1. Calcium Channel Blockers

Although elderly subjects are less sensitive to the effects of verapamil on cardiac conduction, older people do show a greater drop in blood pressure and heart rate in response to a given dose of verapamil [20]. This might be explained by an increased sensitivity to the negative inotropic and vasodilatating effects of verapamil, as well as diminished baroreceptor sensitivity. Diltiazem also shows age-related changes in metabolism, but these changes do not appear to affect blood pressure or heart rate response [24]. The administration of diltiazem as a bolus injection causes greater prolongation of the PR interval (dromotropic effect) in young than in elderly subjects [4].

Dihydropyridines initially have a greater effect on blood pressure in elderly persons, possibly due to an age-related decrease in baroreceptor response. The greater effect may be transient and disappears in about 3 months [20].

#### 3.2.2. Beta-Blocking Agents

Reduced *β*-adrenoreceptor function is observed in advanced age. Both *β*-agonist and *β*-antagonist show reduced responses with age [20]. This is secondary to impaired *β*-receptor function due to variations in receptor confirmation, alterations in binding affinity to the guanine nucleotide subunit (*G*_*s*_), or receptor downregulation. The total number of receptors seems to be maintained but the postreceptor events are changed because of alterations of the intracellular environment. The responsiveness of *α*-adrenoreceptors is preserved with advancing age.

### 3.3. Central Nervous System-Active Drugs

Many drugs affecting the central nervous system (CNS) cause an exaggerated response in older persons. Elderly patients are particularly vulnerable to adverse effects of antipsychotics, such as extrapyramidal motor disturbances, arrhythmias, and postural hypotension. Agents with anticholinergic effects can also impair cognition and orientation in patients with a cholinergic deficit such as those with Alzheimer's disease. Advanced age is also associated with increased sensitivity to the central nervous system effects of benzodiazepines. Postural sway is increased and patients are more likely to lose their balance after triazolam administration [25]. The sedative effects of midazolam are much stronger with the regular given dose [26]. The exact mechanisms responsible for the increased sensitivity to these drugs with ageing are unknown. However, drugs may penetrate the CNS more readily with advancing age. For example, functional activity of the P-glycoprotein efflux pump in the blood-brain barrier is reduced by aging [27]. Reported differences for the benzodiazepines could be due to differences in drug distribution to the CNS.

Anaesthetic agents generally show an increase in sensitivity in the elderly. For example, propofol sensitivity increases with age [28]. Neuromuscular blockers do not show increased sensitivity, lower dosing requirements are primarily due to altered pharmacokinetics [28]. Sensitivity of opioids increases by about 50% in elderly individuals [29, 30].

## 4. Variability in Response to Medicines

Older people display considerable variability in responses to medicines, as well as beneficial effects as adverse effects [31]. Patients may benefit from antipsychotics for delirium and behavioural and psychological symptoms in dementia. Many other antipsychotics do not show benefit, but do have adverse effects [32]. About half of the patients treated with haloperidol suffer extrapyramidal motor disturbances, independent from daily dosage or serum haloperidol concentration [33]. A change in pharmacogenetic factors was not present. Another example is the variable response on anticoagulants. VKAs are associated with a significant risk of adverse outcomes leading to hospitalization in older people. Age, weight, and genotype of pharmacokinetic (CYP2C9) and pharmacodynamic (VKORC1) determinants account for about 60% of the variability in warfarin dose requirements [33–35]. The variability in drug response is multifactorial and the consequence of changes in organ-function, body composition, postreceptor response, homeostatic reserve, and comorbid disease [36, 37]. Also, pharmacogenetic factors may play a role. Frailty is increasingly recognized as a phenotype that is predicitve of adverse health outcomes in older people [38]. Inflammation associated with frailty has the potential to significantly alter drug transporter and metabolizing enzyme expression contributing to variability in drug clearance [39]. Changes in gene expression involve a very small fraction of genes [40]. All in all, the variabilities in responses to medicines are unlikely to have a strong pharmacogenic component [31].

## 5. Medication Use in Elderly Patients

Elderly patients often suffer from several chronic disorders and consequently use more drugs than any other age group. The diminished physiological reserve associated with ageing can be further depleted by acute or chronic disease states and effects of drugs. In most developed countries, about 2/3 of the population ≥65 years take prescription and over the counter (OTC) drugs. At any given time, an average elderly person uses 4-5 prescription drugs and two OTC drugs and fills 12–17 prescriptions a year [4]. The frail elderly patient uses often more than five different drugs. The nursing home resident in The Netherlands receives at least 7-8 different drugs. The mean number of drugs used at admission to a geriatric department was found to be ten [8]. On top of these patients used at mean two OTC medicines. The type of drug used varies with the setting. Nursing home residents use antipsychotics and sedative-hypnotics most commonly, followed by diuretics, antihypertensives drugs analgesics, cardiac drugs, and antibiotics. Psychoactive drugs are prescribed for ~65% of nursing home patients and for ~55% of residential care patients; ~7% of patients in nursing homes receive ≥3 psychoactive drugs concurrently. Community patients use analgesics, diuretics, cardiovascular drugs, and sedatives most often. Older people use OTC medicines to treat minor complaints such as pain, constipation, colds and gastrointestinal symptoms [41]. The most commonly used OTCs are, paracetamol, NSAIDs, antihistamines and drugs for gastric complaints like H_2_ receptor antagonists and protonpump inhibitors. In several countries statins and proton-pump inhibitors are also available as OTC drugs. There are concerns regarding the safety of OTC medicines, especially in elderly patients. In particular, sedatives may increase the risk of falls. The use of multiple medications increases the risk of drug-drug interactions and adverse effects. The varying degrees of hepatic and renal impairment and the potential for a larger pharmacodynamic effect of sedatives in old people can make OTC medicines, even with low doses, harmful. Cebollero-Santamaria et al. [42] showed that bleeding from a peptic ulcer was associated with use of NSAIDs in 81% of 84 patients and that 95% had purchased their NSAIDs as a OTC drug. The use of recommended doses of the OTC NSAIDs has a relatively good safety profile compared to prescription NSAIDs. However patients may take higher doses for a longer period without a gastroprotective drug with serious gastrointestinal toxicity as result [43]. Many older people use OTC drugs to improve their sleep. The risks associated with this use have not been examined [41]. Older people are not always aware of adverse drug reactions (ADRs) caused by OTC H_2_ receptor antagonists, such as confusion, and OTC statins, such as liver and skeletal muscle toxicity.

Documentation of OTC medicines in medical records is uncommon. Only 5% of OTC drugs, used by patients prior to and during hospitalization, were recorded on drug charts [44]. Asking elderly patients, especially those admitted to hospitals, for their use of OTC drugs is important to prevent double-prescription and clinically relevant drug-drug interactions. Not only NSAIDs and antihistamines may cause these interactions, but also herbal drugs as St. John's wort. St. John's wort is used to treat depressive symptoms. In Table 2 clinical important interactions of St. John's wort are summarized [7]. The increasing availability of OTC drugs clearly has benefits. Nevertheless, prescribers must always pay close attention to concomitant OTC medication use in order to minimize adverse drug reactions.

## 6. Polypharmacy versus Appropriate Prescribing

Many drugs benefit elderly patients. Some can be life-saving (i.e., antibiotics and thrombolytic therapy). Oral hypoglycemic agents can improve independence and quality of life while controlling chronic disease. Antihypertensive drugs and influenza vaccines can help prevent or decrease morbidity. Analgesics and antidepressants can control debilitating symptoms. Therefore, appropriateness, that is whether the potential benefits outweigh the potential risks, should guide therapy. Polypharmacy is often defined as the concurrent use of five or more different drugs. The main reasons for polypharmacy are longer life expectancy, multimorbidity and the implementation of evidence-based guidelines [45]. However, polypharmacy also has important negative consequences. Inappropriate polypharmacy contributes to unwanted and often preventable clinically relevant drug-drug and drug-disease interactions as well as adverse drug reactions (ADRs). One-year incidence of potentially inappropriate medication use of frail elderly was found to be 42,1% [46]. Approximately 12% of elderly patients in hospitals are admitted because of ADRs [47]. It is estimated that over half of these ADRs are preventable [21, 48, 49]. Multiple drug use in itself is not necessarily undesirable. The term appropriate prescribing addresses the problems of both inappropriate use of medication as well as inappropriate nonuse of medication (or undertreatment). Comprehensive geriatric assessment and medication review are effective methods to optimize polypharmacy and should comprise both inappropriate use as well as undertreatment [12, 45]. It has been proven that pharmacists and geriatricians may play an important role to optimize polypharmacy in elderly [50, 51].

## 7. Medication Review

Medication review is an essential process in the management of patients with chronic disease. The medication reconciliation process aims to reduce medications errors and consists of four steps [52]. The first step is verification, that is, the list of medications currently used is assembled. The second step is clarification and evaluation: each medication (including formulation and dosage) is checked for appropriateness. The third step is reconciliation: comparison of newly prescribed medications to the old ones and the documentation of the changes. The final step is transmission, in which the updated list is communicated to the next care provider. Medication reconciliation reduced by 43% of the patients adverse drug events (ADEs), which were caused by admission prescribing changes classified as errors, but did not reduce ADEs caused by all admission prescribing changes [53].

Several methods have been developed to assess the appropriateness of drugs prescribed to elderly patients, and methods can be divided into implicit and explicit methods [54, 55]. In an implicit method, medical knowledge and information from the patient are used to determine if a therapy is appropriate. Examples of validated, implicit screening tools are the Medication Appropriateness Index and the prescription optimization method [12, 56]. These methods are patient-tailored and provide opportunity to conduct a complete and flexible assessment of individual pharmacotherapy. Since implicit methods depend on patient information, they are capable of detecting nonspecified problems. However, these methods are often time consuming and are dependent on clinical judgment and knowledge of geriatric pharmacotherapy factors that may vary between physicians. This possible lack of knowledge is less relevant with explicit methods. These are more rigid screening tools based on literature review or expert consensus, and specify inappropriate drug combinations or contraindications. Inappropriate medications can be detected in a consistent manner. The structure of these tools makes it possible to incorporate them easily into software packages, and they can be used as so-called “clinical rules.” The most well-known explicit screening tool is the recently updated Beers drug list, which lists medications to be avoided or adjusted in elderly populations in general or in cases of specific morbidity [57]. Similar lists based on the Beers list have been developed in France, Canada and Norway [58–60]. Other examples of explicit screening tools are START (screening tool to alert doctors to the Right Treatment) and STOPP (screening tool of older person's prescriptions), which are system- defined medicine review tools [61–63]. Explicit screening tools have some disadvantages. For example, the inflexible approach can lead to false-positive signals, because individual patient characteristics or preferences are not taken into consideration—a drug may be inappropriate in general but appropriate for a specific patient. Also, false-negative signals may occur, because nonspecified problems are not detected by these methods. Factors such as time until benefit and drug monitoring are not taken into account. Underprescribing, which means that a disease is not treated according to guidelines, cannot usually be detected by these explicit screening tools (except the START screening tool, which is designed to detect underprescribing [61]).

The prescription optimization method (POM) covers all aspects for appropriate prescribing and consists of six questions [12].

1. Which drugs are really used by the patient? What is the degree of patients adherence?
2. Which drugs, that the patient uses, cause adverse effects?
3. Which drugs are necessary for the patient? Does undertreatment exists?
4. Which drugs are not necessary or contra-indicated?
5. Are there clinically relevant interactions?
6. Is the dose and the dose frequency appropriate?

### 7.1. Structured History to Improve Medication Taking in Elderly

The first step of medication reconciliation is to look at the medicines the patient really uses, including the OTC drugs [64]. Usually, the medication list is assembled by an unstructured interview with the patient. Various resources can be used, such as letters by referring physicians, medication vials, or community pharmacy listings. None of these sources has by itself proven to be completely accurate [65]. To provide physicians with a method for medication history taking, recently the structured history taking of medication use (SHIM, Table 3) was developed. SHIM revealed discrepancies in the medication histories of almost all patients. Actual clinical consequences occurred in one out of five patients, and almost half of these consequences are caused by discrepancies concerning nonprescription OTC drugs. SHIM has the potential to prevent these problems and therefore is a successful first step in the medication reconciliation process [8]. Important to note is that taste disturbances can affect adherence in itself as part of the ageing proces, or by taste disturbances induced by drugs [66, 67].

To improve medication adherence in elderly, a combination of educational and behaviour strategies should always be used [68].

The efficacy and safety of medicines is largely determined by adherence. Adherence is defined as the extent to which a person's behaviour, taking medication, following a diet, and/or executing life-style changes, corresponds with recommendations agreed with a health-care provider [69]. Poor adherence to the treatment of chronic disease is a common problem among the elderly [70]. One of the first articles pointing at the lack of adherence was published in 1957; in only 50% of the patients, who were prescribed tuberculostatics, the drug was found in urine [71]. A Cochrane review reported 50% nonadherence in patients using medicines for chronic diseases [72]. Adherence to antihypertensives and statin therapy is often even lower. Within one year of the start of antihypertensives 50% of the patients have stopped using these drugs [73]. The adherence of elderly patients, prescribed statins, is 60% after 3 months, 43% after 6 months, and 26% after 5 years [74].

The consequences of nonadherence are considerable and include hospital admissions (33–60% of drug related hospital admissions) and higher mortality [21, 75]. Even with use of placebo, high adherence had a 3.5 time greater effect on reducing mortality than the overall active treatment with candesartan in chronic heart failure [76]. This finding suggests that high adherence for taking medicines, is associated with high adherence for life-style advice.

The identification of patient nonadherence is important. Factors that contribute to poor adherence are summarized in Table 4 [9].

A systematic review of barriers to medication adherence in the elderly showed patient-related factors as disease-related knowledge, health literacy and cognitive function, drug-related factors such as adverse effects and polypharmacy [70]. Older person's willingness to take medication for cardiovascular disease prevention is highly sensitive to its adverse effects [77]. Also factors as patient-provider relationship and logistical problems to obtaining medications are identified [70].

In general practice nonadherence is often detected by looking in the medicine cupboard at home. Another method makes use of pharmacy refill records comparing the number of dispensed doses with the number of prescribed doses. A very helpful starting point is to ask the patient and family for the problems they encountered with the drug regimen. The patient should not be blamed for poor adherence. A tool for screening patient adherence is the Brief Medication Questionnaire [78]. Other methods for detecting nonadherence are physiological markers, like low heart-rate with use of beta-blockers, or biochemical measurements in blood or urine such as plasma angiotensin converting enzyme assays to monitor ACEI adherence.

Several methods have been shown to improve adherence. The most effective approach is multilevel targeting at several factors with several interventions. However effective interventions are often complex and not suitable for daily practice. Education in self-management of the drug regimen has limited effects. A simple and very effective method is the reduction of dose frequency. The best adherence is found with a dose frequency of once a day (79%), decreasing to 69% with b.i.d., 65% with t.i.d and 51% with q.i.d [79].

Integrating the patient's perspective into treatment plans is considered to be very important. The behaviour of prescribers is changing from a paternalistic one-way style towards concordance to improve adherence [80].

### 7.2. Adverse Drug Reactions

Adverse drug events (ADEs) are an important cause of morbidity and mortality in elderly patients [21, 48, 49]. Nursing home and frail elderly patients appear to be at high risk of ADEs. The risk of ADEs is exponentially rather than linearly related to the number of medicines taken. More than 80% of ADEs causing admission or occurring in hospital are type A, that is, they are dose related, predictable, and potentially avoidable. Antibiotics, anticoagulants, digoxin, diuretics, hypoglycaemic agents, antineoplastic agents, and nonsteroidal anti-inflammatory drugs are mainly responsible for type A ADRs. Type B ADRs (idiosyncratic reactions) are less common but can be associated with serious toxicity. Several drugs cause movement disorders and falls in old persons [81, 82]. An approximately linear relationship between the occurrence of ADRs and the number of drugs taken with an 8,6% increase in the risk of ADRs for each additional drugs is found [83].  Table 5 shows common adverse effects of medicines in the elderly. Medication reconciliation reduced by 43% ADE's caused by admission prescribing changes classified as errors [53]. It is important to ask the patient about adverse drug reactions and, if so, to look at alternatives. When drugs have similar efficacy/safety profiles the least expensive option should be prescribed.

### 7.3. Undertreatment

The next step is to analyze the problems and diseases of the patient and to determine which drugs are indicated. It is important to identify indicated drugs that are missing. Undertreatment is a common reason for inappropriate prescribing. It has been shown that undertreatment is frequent in elderly patients, despite the use of many medicines [84–86]. The most common areas of undertreatment were extracted from literature, and are presented in Table 6. Choudhury et al. [87] concluded that a physician's experience with bleeding events associated with warfarin in patients can cause underprescription of warfarin to other patients.

Kuzuya et al. [88] showed that the incidence of polypharmacy among frail community-dwelling older people is lower in the oldest members (>85 years) due to of underuse of medications for chronic diseases. In one study a clear relationship between polypharmacy and underprescription was found [86]. The probability of underprescription increased significantly with the number of medicines.

It appears that general practitioners (GPs) and specialists are not willing to prescribe more drugs to old frail patients with current polypharmacy (e.g., complexity of drug regimens, fear of ADRs, interactions, and poor adherence). Research has shown that for some medical problems a so-called treatment-risk paradox or risk-treatment mismatch exists meaning that patients who are at highest risk for complications have the lowest probability to receive the recommended pharmacological treatment [89, 90]. The application of clinical practice guidelines (CPGs) to the care of older patients with several comorbid diseases may have undesirable effects and there could be reasons not to treat all problems. Moreover, the evidence of the benefit of CPG application in elderly patients with comorbid disease is lacking. Boyd et al. [91] estimated that if the relevant CPGs were followed a hypothetical patient would be prescribed 12 medications. However, undertreatment may be harmful for the patient. In optimising polypharmacy, attention should be directed not only to overtreatment but also to possible undertreatment. The aim is to enhance appropriate prescribing to patients with comorbid diseases. In making decisions, prescribers should consider the remaining life expectancy, goals of care and potential benefits of medications [92]. The study of Leliveld-van de Heuvel et al. showed that general practitioners often have reasons not to prescribe a medicine that is advised by guidelines [93].

### 7.4. Inappropriate Medicines

The indication for a drug is often based on guidelines. However, even if there is an indication for a drug according to the guidelines, it is possible that in specific cases the guidelines can be discarded. In the elderly time-until-benefit and the life expectancy are important factors to consider [92]. Age by itself is no reason to omit drug therapy. To check for contraindicated drugs a list is provided (Table 7). Unnecessary duplications with other drugs should be looked for [48, 60, 61] as well as methods minimizing adverse drug events in older patients [94, 95].

### 7.5. Drug Interactions

Although around 10% of the general population take more than one prescribed medicine, the incidence of combination therapy is greatest in the elderly, in females, and in those who have had a recent hospital admission. Patients aged over 65 years use on average four prescribed medications. The medicines should be prescribable together. It is important to look at clinically significant drug-drug and drug-disease interactions [12].

A list of common drug interactions in elderly patients is illustrated in Table 8.

Patients should be advised not to drink grapefruit juice or pay attention to ADRs if they are using any of the following drugs:

 Antiarrhythmic agents: quinidine.
 Histamine antagonists: Astemizole, Terfenadine.
 Benzodiazepines: Alprazolam, Diazepam, Midazolam, Triazolam.
 Calcium channel blockers: Diltiazem, Felodipine, Nifedipine, Verapamil, Lercanidipine, Nitrendipine.
 HIV medication: Indinavir, Nelfinavir, Ritonavir, Saquinavir.
 Hormones: Estradiol, Hydrocortisone, Progesterone, Testosterone.
 Immune modulators: Cyclosporine, Tacrolimus.
 Macrolide antibiotics: Claritromycin and erythromycin.
 Statins: atorvastatin, simvastatin.
 Other: Aripiprazole, Buspirone, Dexamethasone, Docetaxel, Domperidone, Fentanyl, Haloperidol, Irinotecan, Propranolol, Risperidone, Salmeterol, Tamoxifen, Taxol, Vincristine, Zolpidem.

For the clinically relevant interactions with St. John's wort see Table 2.

### 7.6. Dose and/or Dose Frequency Adjustment

Consider if the prescribed dose is still correct. In elderly patients serum creatinine may be within the reference limits, while renal function is markedly diminished. The Cockcroft and Gault and/or the Modification of Diet in Renal Disease (MDRD) equations may be helpful for a better estimation of glomerular filtration rate for drugs cleared predominantly renally. However, there are concerns about the validity of these methods in frail elderly. A list of drugs whose dosage should be adjusted in case of decreased renal function is presented in Table 9.

This question also serves to make physicians aware of the possibility to decrease the dose frequency, or to combine drugs in combination preparates in order to improve adherence.

Adherence can be increased in several ways, but most evidence exists for reduction of the number of daily doses [72].

Recently the polypharmacy optimization method (POM) has been incorporated in the Polypharmacy guideline in the Netherlands. The POM has become part, as step 1 and 2, of the structured tool to reduce inappropriate polypharmacy (STRIP). The STRIP consists of five steps:

1. Structured history taking of medication, for example according to SHIM [8].
2. Pharmacotherapeutic analysis consisting of analysis of undertreatment, effectiveness of the used medicines, no longer indicated drugs, presence of adverse drug reactions, presence of clinically relevant interactions, necessity to correct the prescribed dose, presence of problems with the use of the medicines.
3. Setting up a pharmacotherapeutical treatment plan, together by physician and pharmacist.
4. Discuss the pharmacotherapeutical plan with the patient and make definite decisions.
5. Monitor the consequences of the plan and make adaptations if necessary.

## 8. Conclusion

Older persons have a significantly higher disease burden compared with younger adults, and they consume almost half of total drug expenditures. Because of the changes in pharmacokinetics and pharmacodynamics with aging, and the increase risk for ADRs there is a need for more clinical and observational studies in the elderly. Underrepresentation of older patients in clinical trials is still reported and may occur for many reasons, but major factors are believed to be exclusions due to comorbid conditions, the use of concomitant medications, and frail health [96–98]. However, even the best preauthorization study cannot answer all the possible questions. Hence, postauthorization studies and ways to examine clinical practice generated information are needed. This is recognised also by the Food and Drug Administration (FDA) and European Medicine Agency (EMA). The EMA's Committee for Medicinal Products for Human use (CHMP) has established a Geriatric Expert Group, to provide scientific advice on issues related to the elderly. An European Geriatic Medicine Strategy is launched in 2011. Information is available on http://www.ema.europa.eu/. In The Netherlands the Expertise centre Pharmacotherapy in Old Persons is raised to improve effective and as safe as possible pharmacotherapy (http://www.ephor.eu). Adequate information is critical for optimal patient-individualized drug use. A multifaceted approach will be required to improve the evidence base to guide prescribers. Use of the STRIP method may help prescribers to optimize polypharmacy. Specific gerontopharmacology education is needed to teach students and prescribers, how to optimize polypharmacy and prescribe appropriate medication to old persons [99].

## Figures and Tables

**Table 1:** Selected pharmacodynamic changes with ageing.

Drug	Pharmacodynamic effect	Age-related change
Antipsychotics	Sedation, extrapyramidal symptoms	Increased
Benzodiazepines	Sedation, postural sway	Increased
Beta-agonists	Bronchodilatation	Decreased
Beta-blocking agents	Antihypertensive effects	Decreased
Vitamine K antagonists	Anticoagulant effects	Increased
Furosemide	Peak diuretic response	Decreased
Morphine	Analgesic effects, sedation	Increased
Propofol	Anesthetic effect	Increased
Verapamil	Antihypertensive effect	Increased

**Table 2:** Clinically relevant drug interactions with St. John's wort [7].

Drug	Effect of interaction with St. John's wort
Amitriptyline	Steady-state concentration decreased by 22%
Cyclosporine	Steady-state concentration decreased by 52%
Digoxin	Steady-state concentration decreased by 25%
Simvastatin	AUC decreased by 50%
Tacrolimus	Steady-state concentration decreased by 80%
Theophylline	Steady-state concentration decreased by 50%
VKAs	INR 50% lower

AUC: area under plasma concentration time curve.

**Table 3:** Structured history taking of medication use (SHIM) questionnaire (Drenth-van Maanen et al., 2011 [8]; http://www.ephor.eu).

Questions asked per drug on the medication list, provided by the community pharmacist
(1) Are you using this drug as prescribed (dosage, dose frequency, dosage form)?
(2) Are you experiencing any side effects?
(3) What is the reason for deviating (from the dosage, dose frequency, or dosage form) or not taking a drug at all?
(4) Are you using any other prescription drugs, which are not mentioned on this list? (View medication containers)
(5) Are you using non-prescription drugs?
(6) Are you using homeopathic drugs or herbal medicines (especially st. Johns wort)?
(7) Are you using drugs that belong to family members or friends?
(8) Are you using any drugs “on demand”?
(9) Are you using drugs that are no longer prescribed?

Questions concerning the use of medicines

(10) Are you taking your medication independently?
(11) Are you using a dosage system?
(12) Are you experiencing problems taking your medication?
(13) In case of inhalation therapy: What kind of inhalation system are you using? Are you experiencing any problems using this system?
(14) In case of eye drops: Are you experiencing any difficulties using the eye drops?
(15) Do you ever forget to take your medication? If so, which medication, why, and what do you do?

Other

(16) Would you like to comment on or ask a question about your medication?

**Table 4:** Methods of measuring adherence (modified Osterberg and Blaschke [9]).

Methods	Advantages	Disadvantages
Directly observed therapy	Most accurate	Patients can hide pills in mouth and then discard them; impractical for routine use
Biochemical measurement of the medicine or metabolite or measurement of a biological marker	Objective	Variations in metabolism and “white coat” adherence can give a false impression; expensive
Patient questionnaires or self-reports	Simple, inexpensive, most useful in clinical practice	Susceptible to error and distortion
Pill counts	Objective, quantifiable and easy to perform	Data easily altered by the patient (e.g., pill dumping)
Rates of prescription refills	Objective, easy to obtain data	A prescription refill is not equivalent to ingestion of medication; requires a closed pharmacy system
Assessment of the patient's clinical response	Simple; easy to perform	Factors other than medication adherence can affect clinical response
Electronic medication monitors	Precise; results are easily quantified; tracks patterns of taking medication	Expensive for example, MEMs; some requires return visits
Measurement of physiologic markers	Often easy to perform	Marker may be absent for other reasons
Patient diaries	Help to correct for poor recall	Easily altered by the patient
Questionnaire for caregiver, for patients who are cognitively impaired.	Help to correct for poor recall; simple; objective	Susceptible to error and distortion

**Table 5:** Common adverse drug reactions in the elderly.

Medicine	Adverse drug reaction
Anticonvulsants	Drowsiness
Anti-parkinsonic drugs	Hallucinations, postural hypotension
Antipsychotic drugs	Drowsiness, movement disorders and falls
Vitamin K antagonists	Bleeding
Digoxin	Nausea, bradycardia, falls
Lithium	Delirium, nausea, ataxia, drowsiness nephrotoxicity, thyroid disturbances
Opioids	Drowsiness, constipation, falls
Sulfonylurea anti-diabetics	Hypoglycemia, falls
Tricyclic antidepressants	Drowsiness, postural hypotension, movement disorders and falls
Verapamil, diltiazem	Bradycardia, hypotension, constipation, falls

**Table 6:** Common undertreated conditions and advised medication according to guidelines.

Disease or problem	Advised medicines
Angina pectoris	beta-receptor blocking drug
Atrial fibrillation	VKA, when contraindicated acetylsalicylic acid
Cardiovascular disease^1^	in case of oversensitiveness: clopidogrel, prasugrel
Cardiovascular disease + LDL > 2.5	Statin
Cerebral infarction/TIA	Consider antihypertensive treatment, even with normal blood pressure
COPD	Inhalation ipratropium or tiotropium/*β*2-agonists
Corticosteroids used > 1 month	Medication to prevent osteoporosis: bisphosphonates
Depression	Antidepressants: SSRI's or nortriptyline
Diabetes Mellitus	Lipid lowering drugs
Diabetes with proteinuria	ACE-inhibitor
Heart failure	ACE-inhibitor, or AT II antagonist if necessary beta-receptor blocking drug
Hypertension	Anti-hypertensive treatment
Insufficient daylight	Vitamin D3
Myocardial Infarction	Acetylsalicylic acid, ACE-inhibitor, beta-receptor blocking drug
NSAID	Gastric protection with Proton Pump Inhibitors
Opioids	Laxatives
Osteoporosis	Antiosteoporosis drugs
Pain	Analgesics

^1^Cardiovascular disease: by atherothrombotic processes caused clinical manifestations, like myocardial infarction, angina pectoris, cerebral infarction, transient ischaemic attack (TIA), aortic aneurysm, and peripheral arterial vessel disease.

**Table 7:** Conditions with (relatively) contraindicated drugs [10, 11].

Disease or problem	Contraindicated drugs
COPD	Long acting benzodiazepines, non-selective beta-receptor blocking drugs (e.g., propranolol, carvedilol, labetalol, sotalol)
Dementia	Strong acting anticholinergic agents^1^
Heart failure	Verapamil, diltiazem, short acting nifedipine, NSAIDs, rosiglitazone
Lower Urinary Tract Syndrome	Anticholinergic agents^1^
Active peptic ulcer disease, reflux oesophagitis, or gastritis/duodenitis	NSAIDs
Narrow angle glaucoma	Strong acting anticholinergic agents^1^
Constipation	Verapamil, diltiazem, anticholinergic agents^1^
Postural hypotension	Tricyclic antidepressants
Parkinson's disease	Metoclopramide, all antipsychotics except clozapine and quetiapine
Hyponatremia (SIADH)	SSRIs
Falls	Psychoactive drugs

^1^Strong acting anticholinergic drugs: spasmolytics, tricyclic antidepressants, and anticholinergic antiparkinsonic drugs.

**Table 8:** The most common drug interactions in elderly patients [12].

Drug	Interaction	Effect
ACE inhibitors	NSAIDs, Coxibs, potassium-sparing diuretics	Decreased renal function, hyperkalemia
Antidepressants	Enzyme inducers^1^	Less antidepressant effect
Antihypertensives	Vasodilators, antipsychotic drug, tricyclic antidepressants	Increased antihypertensive effect
	NSAIDs	Decreased antihypertensive effect
Beta-receptor-blocking drugs	Anti-diabetic drugs	Masks hypoglycemia
	Fluoxetine, paroxetine (especially in combination with metoprolol and propranolol)	Bradycardia
Corticosteroids (oral)	NSAIDs	Gastro-duodenal ulcer disease
	enzyme inducers^1^	Decreased corticosteroid effect
Digoxin	NSAIDs, diuretics, qinidine, verapamil, diltiazem, amiodarone	Digoxin intoxication
Fluoroquinolones	Al-Mg containing antacids, iron, calcium	Decreased bioavailability
Levodopa	Iron	Decreased bioavailability
Lithium	NSAIDs, thiazide diuretics, antipsychotics	Lithium toxicity
Phenytoin	Enzyme inhibitors^2^	Increased toxicity
Sulfonylurea anti-diabetics	SSRIs, chloramphenicol, VKA's, phenylbutazone	Hypoglycemia
SSRIs	Diuretics, NSAIDs	Hyponatremia, gastric bleeding
Tetracyclines	Antacids, iron	Decreased bioavailability
VKA's	Acetylsalicylic acid, NSAIDs, metronidazole, miconazole and other azole-type drugs	Bleeding

^1^Important enzyme inducers: carbamazepine, rifampicin, phenobarbital, phenytoin, St. John's wort.

^2^Important enzyme inhibitors: verapamil, diltiazem, amiodarone, fluconazole, miconazole, ketoconazole, erythromycin, claritromycin, sulfonamides, cimetidine, ciprofloxacin, and grapefruit juice.

**Table 9:** Adjustment of dosage in renal insufficiency. Calculate the creatinine clearance or GFR (http://nephron.com/cgi-bin/CGSI.cgi). For Crcl < 10 mL/min consult the nephrologist.

	Decreased renal function and dose adjustment
ACE Inhibitors	
Benazepril	Clcr 10–30 mL/min: start with 2.5–5 mg once daily. Adjust dosage based on effect.
Captopril	Clcr 10–30 mL/min: start with 12.5–25 mg once daily. Adjust dosage based on effect until 75–100 mg/day
Cilazapril	Clcr 10–30 mL/min: start with max. 0.5 mg/day. Adjust dosage based on effect until max. 2.5 mg/day
Enalapril	Clcr 10–30 mL/min: start with max. 5 mg/day. Adjust dosage based on effect until max. 10 mg/day
Lisinopril	Clcr 10–30 mL/min: start with max. 5 mg/day. Adjust dosage based on effect until max. 40 mg/day
Perindopril	Clcr 30–50 mL/min: max. 2 mg/day; Clcr 10–30 mL/min: max. 2 mg every two days
Quinapril	Clcr 30–50 mL/min: start with 5 mg/day; Clcr 10–30 mL/min: start with 2.5 mg/day. Adjust dosage based on effect.
Ramipril	Clcr 20–50 mL/min: start with max. 1.25 mg/day. Adjust dosage based on effect.
	Clcr 10–20 mL/min: insufficient data for sound advise
Trandolapril	Clcr 10–30 mL/min: start with max. 0.5 mg/day. Adjust dosage based on effect until max. 2 mg/day
Zofenopril	Clcr 10–50 mL/min: start with max. 7.5 mg/day. Adjust dosage based on effect until max. 15 mg/day

Antibiotics	
Cephalosporins	
Cephalexin	Clcr 10–50 mL/min: prolong interval to once per every 12 hours.
Cephalothin	Clcr 50–80 mL/min 2 g every 6 hours; 30–50 mL/min 1.5 g every 6 hours; 10–30 mL/min 1 g every 8 hours.
Cephamandole	Clcr 50–80 mL/min 2 g every 6 hours, in case of life-threatening infection 1.5 g every 4 hours;
	Clcr 30–50 mL/min 2 g every 8 hours, in case of life-threatening infection 1.5 g every 6 hours;
	Clcr 10–30 mL/min 1.25 g every 6 hours, in case of life-threatening infection 1 g every 6 hours.
Cephazolin	Clcr 30–50 mL/min: 500 mg every 12 hours; 10–30 mL/min: 500 mg every 24 hours.
Cephradine	Clcr <30 mL/min: contra-indicated
Cephtazidime	Clcr 30–50 mL/min: 1 g every 12 hours; 10–30 mL min: 1 g every 24 hours.
Cephtibuten	Clcr 30–50 mL/min: 200 mg every 24 hours; 10–30 mL/min: 100 mg every 24 hours.
Cephuroxime parenteral	Clcr 10–30 mL/min: standard dosage every 12 hours.

Fluoroquinolones	
Ciprofloxacin	Clcr 10–30 mL/min: 50% of normal dosage
Levofloxacin; ofloxacin	Clcr 30–50 mL/min: 50% of normal dosage; Clcr 10–30 mL/min: 25% of normal dosage
Norfloxacin	Clcr 10–30 mL/min: prolong interval to once every 24 hours

Nitrofurantoin	
Nitrofurantoin	Clcr < 50: contra-indicated. Risk of neuropathy and failure of therapy.

Macrolide	
Claritromycin	Clcr 10–30 mL/min: 50% of normal dosage with normal dose frequency

Penicillins	
Amoxicillin/clavulanate	Clcr 10–30 mL/min: standard dosage every 12 hours (orally, i.v. of.im.)
Benzylpenicillin	Clcr 10–30 mL/min: dosage dependent of indication. Consider intended effect, risks of overdosage and underdosage.
Piperacillin	Clcr 30–50 mL/min: max. 12 g per day in 3 or 4 doses; Clcr 10–30 mL/min: max. 8 g per day in 2 doses
Piperacillin/tazobactam	Clcr 30–50 mL/min: piperacillin/tazobactam 12 g/1.5 g per day in 3 or 4 doses Clcr 10–30 mL/min: piperacillin 4 g/0.5 g every 12 hours

Tetracyclines	
Tetracycline	Clcr 10–30 mL/min: maintenance dosage 250 mg once daily

Antidiabetics	
Metformin	Clcr 30–50 mL/min: start with twice daily 500 mg; Clcr 10–<30 mL/min: contraindicated
Sulfonylurea (e.g., Tolbutamide)	Clcr < 50 mL/min start with half the dosage

Antihistaminics	
Acrivastine	Clcr 10–50 mL/min: 50% of normal dosage OR prolong interval to 1-2x per day
Cetirizine/Levocetirizine/Hydroxyzine/ Fexofenadine/Terfenadine	Clcr 10–50 mL/min: 50% of normal dosage

Antimycotics	
Fluconazole	In case of >once daily dosing regimen: Clcr 10–50 mL/min: normal starting dosage, decrease maintenance dosage until 50% of normal dosage
Flucytosine	Clcr 30–50 mL/min: prolong interval to once every 12 hours, then based on serum plasma concentration
	Clcr 10–30 mL/min: prolong interval to once every 24 hours, then based on serum plasma concentration
Terbinafine	Clcr 10–50 mL/min: 50% of normal dosage

Antiparkinson drugs	
Pramipexole	Clcr 30–50 mL/min: start with 0.125 mg (= 0.088 base) twice daily, then based on effect/adverse events
	Clcr 10–30 mL/min: start with 0.125 mg (= 0.088 base) once daily, then based on effect/adverse events

Antithrombotics	
Dabigatran	Clcr <30 mL/min: contra-indicated
Eptifibatide	Clcr 10–50 mL/min: normal starting dosage, then 50% of normal dosage
Tirofiban	Clcr 10–30 mL/min: 50% of normal dosage

Antiviral medication	
Acyclovir orally	Decrease dosage used for herpes zoster treatment: Clcr 10–30 mL/min: 800 mg 3 times a day
Amantadine	Start with 200 mg, maintenance dosage: Clcr 50–80 mL/min: 100 mg once daily;
	Clcr 30–50 mL/min: 100 mg every 2 days; Clcr 10–30 mL/min 100 mg every 3 days.
Cidofovir	Clcr <50 mL/min: preferably do not use
Famciclovir	Clcr 30–50 mL/min: normal dosage every 24 hours; 10–30 mL/min: 50% of normal dosage every 24 hours
Foscarnet	Clcr 30–80 mL/min: dosage according to schedule manufacturer; <30 mL/min: do not use
Ganciclovir	Induction: Clcr 50–80 mL/min: 50% of normal dosage every 12 hours; 30–50 mL/min: 50% of normal dosage every 24 hours; 10–30 mL/min: 25% of normal dosage every 24 hours
	Maintenance: Clcr 50–80 mL/min: 50% of normal dosage every 24 hours; 30–50 mL/min: 25% of normal dosage every 24 hours; 10–30 mL/min: 12.5% of normal dosage every 24 hours
Oseltamivir	Clcr 10–30 mL/min: 50% of normal dosage OR normal dosage but double interval
Ribavirin	Clcr 10–50 mL/min: dosage based on hemoglobin concentration
Valaciclovir	Clcr 10–80 mL/min: adjust dosage according to schedule manufacturer
Valganciclovir	Clcr 30–50 mL/min: 50% of normal dosage plus double interval
	Clcr 10–30 mL/min: 50% of normal dosage twice a week

Beta-receptor-blocking drugs	
Acebutolol; Atenolol	Clcr 10–30 mL/min: 50% of normal dosage
Bisoprolol	Clcr 10–20 mL/min: start with 50% of normal dosage. Then max. 10 mg/day
Sotalol	Clcr 30–50 mL/min: max 160 mg/day; Clcr 10–30 mL/min: max. 80 mg/day

Calcium antagonists, dihydropyridine type	
Barnidipine	Clcr <50 mL/min: contraindicated

Digoxin	
Digoxin	Clcr 10–50 mL/min: decrease initial dosage by 50%, then go to 0.125 mg/day. Next adjust dosage based on clinical symptoms

DMARDs	
Anakinra	Clcr < 30 mL/min: contraindicated
Methotrexate	Clcr 40–70 mL/min: 50% of normal dosage. Clcr < 40 mL/min: based on serum plasma concentration

Gout medication	
Allopurinol	Clcr 50–80 mL/min: 300 mg/day; 30–50 mL/min: 200 mg/day; 10–30 mL/min: 100 mg/day
Benzbromarone	Clcr <30 mL/min: contraindicated
Colchicine	Clcr 10–50 mL/min: 0.5 mg/day

H_2_-antagonists	
Nizatidine; cimetidine; famotidine; ranitidine	Clcr 10–30 mL/min: 50% of normal dosage, once daily

Hypnotics, sedative agents, anxiolytic drugs, Antipsychotics	
Chloralhydrate	Clcr <50 mL/min: preferably do not use
Meprobamate	Clcr 10–50 mL/min: 50% of normal dosage OR double dosage interval
Risperidone	Clcr 10–50 mL/min: 50% of normal dosage, then based on effect and adverse events

Muscle relaxants	
Baclofen	Clcr 10–50 mL/min: start with 5 mg once daily, then adjust based on effect and adverse events.
Tizanidine	Clcr 10–30 mL/min: start with 2 mg once daily, then increase dosage slowly based on effect and adverse events. End with increasing the dose frequency.
NSAIDs	All NSAID's: Clcr < 30 mL/min: consider if chronic use is indicated. Check renal function previously to and 1 week after start

OPIOIDs	
Morphine	Clcr 10–50 mL/min: dosage based on effect and adverse events. Be alert to accumulation of M6G
Tramadol	Clcr 10–30 mL/min: decrease dose frequency to 2-3 x per day In case of retard tablet max. 200 mg per day

Tuberculostatics	
Ethambutol	Clcr 10–50 mL/min: 50% of normal dosage

Vertigo medication	
Piracetam	Clcr 30–50 mL/min: 50% of normal dosage; Clcr 10–30 mL/min: 25% of normal dosage

Xanthine derivates	
Pentoxifylline	Clcr 30–50 mL/min: 400 mg twice daily; Clcr 10–30 mL/min: 400 mg once daily
